# Hongjingtian Injection Attenuates Myocardial Oxidative Damage via Promoting Autophagy and Inhibiting Apoptosis

**DOI:** 10.1155/2017/6965739

**Published:** 2017-07-19

**Authors:** Shujing Zhang, Ling Zhang, Han Zhang, Guanwei Fan, Jiuwen Qiu, Zongbao Fang, Haibo Wu, Yi Wang, Xiaoping Zhao

**Affiliations:** ^1^College of Pharmaceutical Sciences, Zhejiang University, Hangzhou 310058, China; ^2^Department of Cardiology, Second Affiliated Hospital, College of Medicine, Zhejiang University, Hangzhou 310009, China; ^3^Key Laboratory of Pharmacology of Traditional Chinese Medicine Formulae, Ministry of Education, Tianjin University of Traditional Chinese Medicine, Tianjin 300193, China; ^4^Tonghua Yusheng Pharmaceutical Co. Ltd., Tonghua 134008, China; ^5^College of Preclinical Medicine, Zhejiang Chinese Medical University, Hangzhou 310053, China

## Abstract

Natural products with antioxidative activities are widely applied to prevent and treat various oxidative stress related diseases, including ischemic heart disease. However, the cellular and molecular mechanisms of those therapies are still needed to be illustrated. In this study, we characterized the cardioprotective effects of Hongjingtian Injection (HJT), an extensively used botanical drug for treating coronary heart disease. The H/R-induced profound elevation of oxidative stress was suppressed by HJT. HJT also attenuates oxidative injury by promoting cell viability, intracellular ATP contents, and mitochondrial oxygen consumption. Validation experiments indicated that HJT inhibited H/R-induced apoptosis and regulated the expression of apoptosis-associated proteins Bcl-2 and cleaved caspase3. Interestingly, HJT significantly regulated the expression of autophagy-related proteins LC3, Beclin, and mTOR as well as ERK and AKT. We provide evidence that the mechanism involves activation of AKT/Beclin-1, AKT, and ERK/mTOR pathway in cardiomyocyte autophagy. Histological and physiological evaluation revealed that HJT significantly decreased the infarct area of the heart, improved cardiac function, and increased the expression of LC3B in a rat model of coronary occlusion. From the obtained data, we proposed that HJT diminished myocardial oxidative damage through regulating the balance of autophagy and apoptosis and reducing oxidative stress.

## 1. Introduction

In the recent years, there has been a growing recognition of the importance of oxidative stress to pathogenic progress of various chronic diseases, as well as for the development of novel therapies [1]. As an important resource for alternative and complementary therapy, natural products contain various kinds of bioactive compounds with antioxidant capacity [2, 3]. However, the precise mechanism of action of antioxidant natural products is poorly understood. Hongjingtian Injection (HJT), a botanical drug manufactured from extracts of *Rhodiola wallichiana* var. *cholaensis*, has a long history of use in China for the prevention and management of various vascular diseases such as angina pectoris and coronary heart disease [4]. Modern analytical techniques identified the major components of HJT including ferulic acid, lotaustralin, salidroside, and its derivatives [5]. Recent biological and pharmacological investigations also showed that HJT can dilate the coronary artery and reduce cardiac afterload in dog [6]. The extracts of *Rhodiola rosea* L., another medicinal plant in Eastern Europe which contains similar chemical constituents of HJT, are regarded to stimulate the nervous system; enhance physical and mental performance; and treat fatigue, psychological stress, and depression [7]. Recent reports showed that salidroside, an active ingredient of the root of *Rhodiola rosea* L., possessed a range of pharmacological properties, including antihypoxia, anti-inflammation, antioxidative, anticancer, antiaging, and neuroprotective effects [8–12], especially the therapeutic action of salidroside in cardioprotective effects which has been well documented in the laboratory [13]. Although HJT exerts a large variety of biological activities, such as promoting the activities of antioxidant enzymes [14], increasing tolerance to hypoxia [11], an unclear pharmacological mechanism of action, has become a common problem faced in clinical application.

Autophagy is a conserved cellular degradation system involving the rearrangement of subcellular membranes to sequester cytoplasms and organelles for delivery to the lysosomes where degrading and recycling damaged or unnecessary cytoplasmic contents [15]. Therefore, autophagy is usually activated by starvation-induced signals and plays an important role in cellular response to nutrient deprivation [16]. Recently, autophagy is well reported to be an important regulator of many cardiovascular diseases such as myocardial ischemia/reperfusion injury [17–19]. In addition, it has been shown that there is a crosstalk between autophagy and apoptosis in heart disease [20]; for example, reactive oxygen species not only trigger apoptosis but also are essential for autophagy [21, 22].

In this study, we examined the cardioprotective effects of HJT against oxidative stress both in H/R-induced myocardial injured cells and in myocardial ischemia-injured rats and hypothesized that the protection mechanism of HJT might be associated with the enhancement of the antioxidant system. We validated HJT enhanced autophagy to promote the survival of cardiomyocytes from oxidative stress damage both in vitro and in vivo, as well as inhibit apoptosis in cells subjected to H/R injury.

## 2. Materials and Methods

### 2.1. Cell Culture and Hypoxia-Reoxygenation Model

The H9c2 cardiomyocytes were obtained from the cell bank of the Shanghai Institutes for Biological Sciences, cultured in 4.5 g/l glucose DMEM (Corning Cellgro) supplemented with 10% fetal bovine (GIBCO), 100 U/ml penicillin, and 100 *μ*g/ml streptomycin. H9c2 cardiomyocytes were seeded in 6-well plates, 96-well plates, or other types of plates and were incubated for 24 h at 37°C in an atmosphere of 5% CO_2_. For the hypoxia-reoxygenation (H/R) model, H9c2 cardiomyocytes were rinsed once with PBS buffer and then refreshed with glucose-free DMEM (GIBCO) (pre-eliminated oxygen with 95% N_2_). Cells were then immediately placed into a sealed chamber loaded with mixed gas containing 5% CO_2_ and 95% N_2_. H9c2 cardiomyocytes were incubated at 37°C for 1 h, 2 h, 6 h, 12 h, or 24 h before reperfusion up to different experimental objectives. For reperfusion, H9c2 cardiomyocytes were refreshed with glucose-containing DMEM supplemented with fetal bovine and continued to be incubated at 37°C in an atmosphere of 5% CO_2_ for 2 h. Cells were divided into three groups: control, H/R, and H/R + HJT. Control cells were always incubated in glucose-containing DMEM supplemented with fetal bovine at 37°C in an atmosphere of 5% CO_2_. In the group of H/R + HJT, HJT injection with different concentrations (diluted 75, 100, and 200 times) were dissolved in glucose-free DMEM and added to the cells at the onset of hypoxia.

### 2.2. The Scavenging of DPPH Radical, Malondialdehyde (MDA), and Reactive Oxygen Species (ROS) Assay

The scavenging of DPPH radical and the content of MDA and ROS were measured according to the instructions of commercial assay kits. DPPH radical was scavenged by HJT injection with different concentrations (diluted 1600,800,400, 200, 100, 50, 25, and 12.5 times). MDA assay kit (Beyotime Institute of Biotechnology) was used to detect the MDA content of H9c2 cardiomyocytes. The level of intracellular ROS was determined by the reactive oxygen species assay kit (Beyotime Institute of Biotechnology) and observed by fluorescence microscopy. Cellular fluorescence was analyzed with ImageJ. The H9c2 cardiomyocytes for ROS assay were kept in a chamber for 1 h while MDA assay for 24 h.

### 2.3. Cell Viability, ATP, and Mitochondrial Oxygen Consumption Rate Assay

Cell viability, the level of ATP, and the mitochondrial oxygen consumption rate in H9c2 cardiomyocytes were measured according to the instructions of commercial assay kits. Cell viability was assessed by 3-(4,5-dimethylthiazol-2-yl)-2,5-diphenyltetrazolium bromide (MTT) assay after the designated treatment. ATP content was assessed by CellTiter-Glo Luminescent Cell Viability Assay kit (Promega). Cell respiration was measured using a Liquid-Phase Oxygen Measurement System (Hansatech, England), and mitochondrial respiration was quantified according to the oxygen consumption rate.

### 2.4. Detection of Cell Apoptosis by Flow Cytometry

Apoptosis was determined by Annexin V-FITC/PI apoptosis assay kit. Double staining with FITC-Annexin V and PI was carried out as per manufacturer's instructions. Briefly, after the designated treatment (24 h hypoxia/2 h reoxygenation), cells were harvested and washed twice in ice-cold PBS. Then, Annexin V-FITC and propidium iodide (PI) were added to each sample for 15 min at 37°C in the dark. Cellular fluorescence was analyzed with a flow cytometer (BD Accuri C6).

### 2.5. Western Blot Analysis

Protein sample preparation and Western blot analysis were performed using standard procedures. In brief, 1 × 10^6^ H9c2 cardiomyocytes were seeded in 6-well plates. At the end of the designated treatment, H9c2 cardiomyocytes were rinsed twice with precooled PBS buffer and gently lysed with a lysis buffer for Western blot and IP on ice to extract the protein. BCA assay was used to measure the protein concentration. The protein was separated by SDS-PAGE gel electrophoresis and transferred onto PVDF membrane. Membrane was blocked with 5% BSA for 2 h at room temperature. After being washed with TBST, the membrane was incubated with primary antibody overnight at 4°C and then the membrane was incubated with an appropriate HRP-conjugated secondary antibody at room temperature for 1 h. The antigen-antibody complexes were then detected with an ultra-sensitive-enhanced chemiluminescent (ECL) substrate (Invitrogen) then visualized by GelDoc XR System (BIO RAD). GAPDH and *β*-actin were used as internal standards.

### 2.6. Confocal Microscopy and Transmission Electron Microscopy (TEM)

After designated treatments (12 h hypoxia/2 h reoxygenation), H9c2 cardiomyocytes were loaded with 1 *μ*M MitoTracker Green (Molecular Probes) and LysoTracker Red (Molecular Probes) at 37°C for 30 min. Cellular fluorescence was observed on a confocal microscope. Cell samples (with the treatment of 1 h hypoxia/2 h reoxygenation) were processed for TEM assay according to routine procedures. Autophagosomes with a double membrane in cardiomyocytes were observed by the use of an H-7650 TEM.

### 2.7. Animals and Experimental Protocols

Male Sprague-Dawley rats (220–240 g) were purchased from Beijing Vital River Laboratory Animal Technology Co. Ltd. For the myocardial ischemia (MI) model, rats were anesthetized by intraperitoneal injection of pentobarbital (50 mg/kg). Cardiac electrophysiology was dynamic monitored with the use of an electrocardiogram machine during the model-making period. The pericardium was opened to expose the heart after a left thoracotomy. A 4–0 silk suture was ligatured around the left anterior descending coronary artery (LAD) 3–4 mm from its origin. Then, the heart was returned to the chest and the incision on the chest was closed. The sham-operated rats underwent the same surgical procedures but without LAD ligation. Successful MI rats were randomly divided into three groups (*n* = 10 in each group) for treatment: model, low-dose HJT (low-HJT; 0.2 ml/100 g), and high-dose HJT (high-HJT; 0.4 ml/100 g). Both the treatments were given once daily with caudal vein injection consecutively for seven days while the sham and model groups received an equivalent saline.

### 2.8. Echocardiography and Hemodynamic Measurements

M-mode echocardiography and pulsed-wave Doppler echocardiography of mitral inflow were performed with the use of Vevo 2100 imaging system (Vevo, Canada) and multipurpose polygraph (MP-150, biopac). The left ventricular (LV) ejection fraction (EF, %), fractional shortening (FS, %), LV volume (LV Vol; s, *μ*l), and average peak velocity (Peak Vel, mm/s) were measured as indicators of cardiac function.

### 2.9. Infarct Area Measurement and Immunohistochemistry Assays

At the end of the experiment, rats were anesthetized by intraperitoneal injection of pentobarbital (50 mg/kg) and rat hearts were quickly removed. Hearts were cleaned in the saline and cut into five pieces on average. Slices were incubated at 37°C in 1% TTC solution for 5 min. TTC stains viable tissue bright red while the infarct area remains grayish-white. Slices were photographed with a digital camera. Calculated the infarction area ratio of total slice area by Image Plus software.

Other hearts were harvested for immunohistochemistry assays. Immunohistochemistry was performed to detect the marker of autophagy after myocardial ischemia. Hearts were fixed in 10% formalin and were paraffin embedded. The paraffin-embedded myocardial tissues were sectioned and incubated with a monoclonal antibody for the LC3B protein. Positive staining (brown yellow) was identified under a light microscope.

### 2.10. Statistical Analysis

All values are expressed as the means ± SD. One-way ANOVA was used to analyze differences among the groups. Statistical analysis was performed using GraphPad Prism. *P* values of less than 0.05 were considered statistically significant.

## 3. Results

### 3.1. HJT Injection Reduces H/R-Induced Oxidative Stress in H9c2 Cardiomyocytes

**Figure 1:**
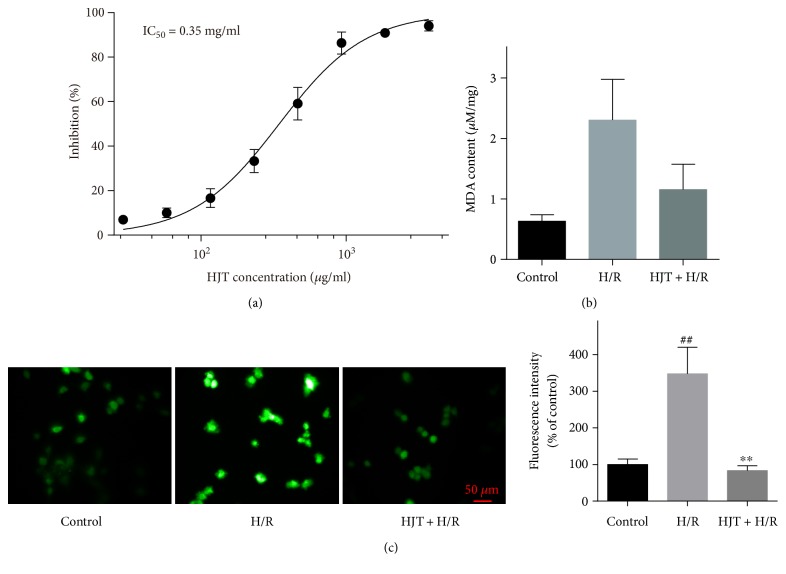
HJT suppressed oxidative stress in the H9c2 cardiomyocytes suffering from H/R. (a) Scavenging of DPPH radical by HJT with different concentrations. (b) Effects of HJT injection on the content of MDA (c) Fluorescent microscopy of H9c2 cardiomyocytes stained with H_2_DCFDA after exposure to 1 h hypoxia and 1 h reoxygenation with or without HJT treatment, scale bar, 50 *μ*m. ^##^*P* < 0.01 versus the control group without H/R, and ^∗∗^*P* < 0.01 versus the group treated with H/R alone.

Scavenging of 1,1-diphenyl-2-picryl-hydrazyl (DPPH) radical has been regarded as the standard assay for evaluation antioxidant activity of natural product [23]. To verify the antioxidative activity of HJT, DPPH radical scavenge assay was applied to determine HJT samples with different concentrations. The result indicated that HJT exhibited satisfied effect on clearing the oxygen free radical with IC_50_ of 0.35 mg/ml (Figure 1(a), the solid content of HJT is 46.47 mg/ml).

We further evaluated the cardioprotective effects of HJT in cardiomyocytes with H/R injury. Malondialdehyde (MDA) as biomarkers of oxidative stress was measured to evaluate oxidative damage [24]. As shown in Figure 1(b), the H/R treatment increased intracellular MDA content in H9c2 cardiomyocytes compared with that in the control group, and the HJT treatment reversed the change. In addition, mitochondrial oxidative stress was often caused by increased intracellular reactive oxygen species (ROS) formation. ROS are frequently induced by ER stress to synergistically trigger autophagy and apoptosis [25]. Therefore, the accumulation of H/R-induced ROS was measured by fluorescence microscope. As expected, the H/R treatment induced high levels of ROS in H9c2 cardiomyocytes, and HJT served as a ROS scavenger reducing H/R-induced ROS production.

### 3.2. HJT Increased Cell Viability and ATP Level and Upregulated Mitochondrial Oxygen Consumption in H/R-Induced H9c2 Cardiomyocytes

**Figure 2:**
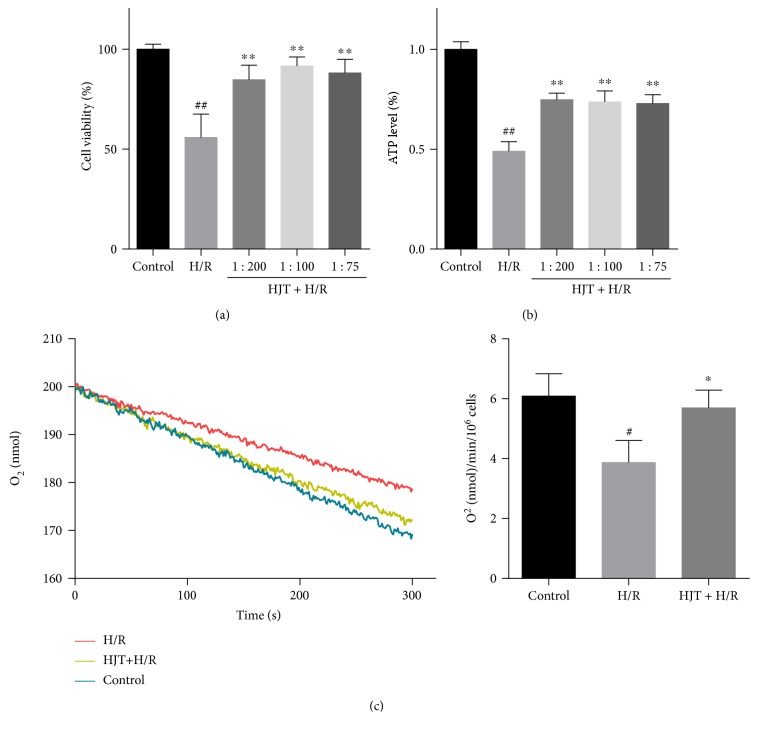
HJT decreased cell death and strengthened the function of mitochondrial. (a-b) Cell viability ((a), 24 h H/2 h R) and ATP level ((b), 12 h H/1 h R) were determined by MTT assay and CTG assay. (c) Effects of HJT injection on mitochondrial oxygen consumption rate. Representative curves of oxygen consumption recorded by the Clark-type oxygen electrode, and H/R-injured cells were preincubated with HJT. Results were obtained from three independent experiments. ^#^*P* < 0.05, ^##^*P* < 0.01 versus the control group without H/R, ^∗^*P* < 0.05, and ^∗∗^*P* < 0.01 versus the group treated with H/R alone.

Mitochondria are the power manufacturing factories of cells that function in cardiac energy metabolism and play an important role in the process of H/R-induced cardiomyocyte apoptosis [26]. Hypoxia results in a series of changes in the structure and function of mitochondria, including impaired ATP synthesis, increased mitochondrial proton leakage, and especially the burst of ROS generation which was determined above [27]. Our experiments showed that the cells were effectively protected by HJT at different concentrations from H/R injury (Figure 2(a)). As a specific parameter of mitochondrial function, intracellular ATP content in cardiomyocytes exposed to oxidative stress were measured. After cells were exposed to 12 h of hypoxia followed by 1 h reoxygenation, the content of ATP was significantly decreased, while the HJT treatment led to the recovery of ATP content (Figure 2(b)). The effects of HJT on mitochondrial function were further investigated by measuring cellular respiration in H9c2 cardiomyocytes [28]. As shown in Figure 2(c), basal respiration of H/R-treated cardiomyocytes was significantly dropped compared with that of the control group. Preincubation of HJT attenuated the decrease of oxygen consumption significantly (6.09 ± 0.61, 3.87 ± 0.61, and 5.69 ± 0.49 nmol O_2_/ml/min in the control, H/R, and HJT-treated cells, resp.). These results suggested that HJT promoted cell survival and strengthened the function of mitochondria.

### 3.3. HJT Attenuated H/R-Induced Apoptosis in H9c2 Cardiomyocytes

**Figure 3:**
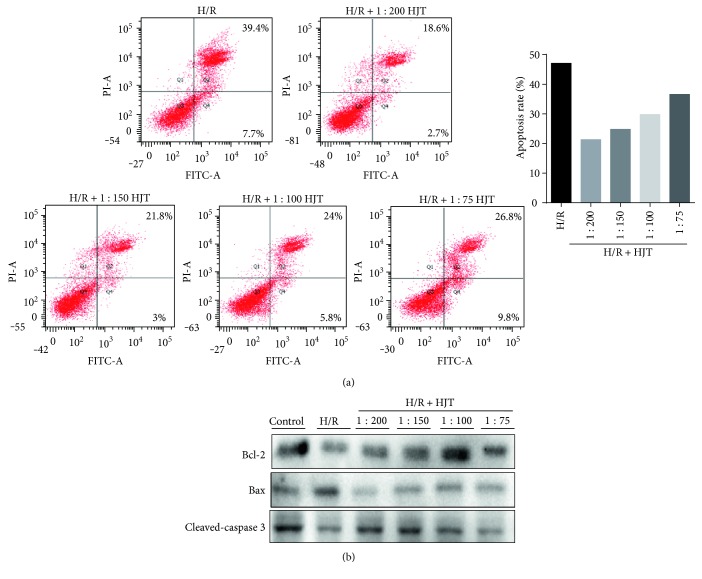
HJT inhibited apoptosis. (a) H/R-induced cell apoptosis rate was quantified by flow cytometry. (b) Western blot analysis of apoptosis-related proteins: Bcl-2, Bax, and cleaved caspase 3 in the cell groups.

Evidences showed that antioxidants help cardiomyocytes resist H/R-induced oxidative damage and apoptosis, which has become a novel therapeutic strategy in the treatment of H/R injury [29]. Thus, we first examined the apoptosis rate of H9c2 cardiomyocytes after treatment with HJT by flow cytometry. Exposure of H9C2 cardiomyocytes to H/R led to an increase in apoptosis rate, whereas treatment with HJT decreased apoptosis rate (Figure 3(a)).

On the basis of the results of the previous step, apoptosis-related proteins were chosen for further investigation. Apoptosis is regulated by apoptosis-regulating proteins, which are divided into two major categories: proapoptotic proteins (e.g., Bax) and antiapoptotic proteins (e.g., Bcl-2). In addition, caspase family acts as the initiator and executor of apoptosis; for instance, caspase-3 is one of the important markers of apoptosis [30]. As shown in Figure 3(b), H/R resulted in a decrease in cleaved caspase 3 expression and Bcl-2/Bax ratio. The HJT treatment increased the levels of leaved caspase 3 and Bcl-2/Bax ratio. These data suggested that another pathway might be involved in apoptosis regulated by HJT.

### 3.4. HJT Facilitates Autophagy in H/R-Induced H9c2 Cardiomyocytes

**Figure 4:**
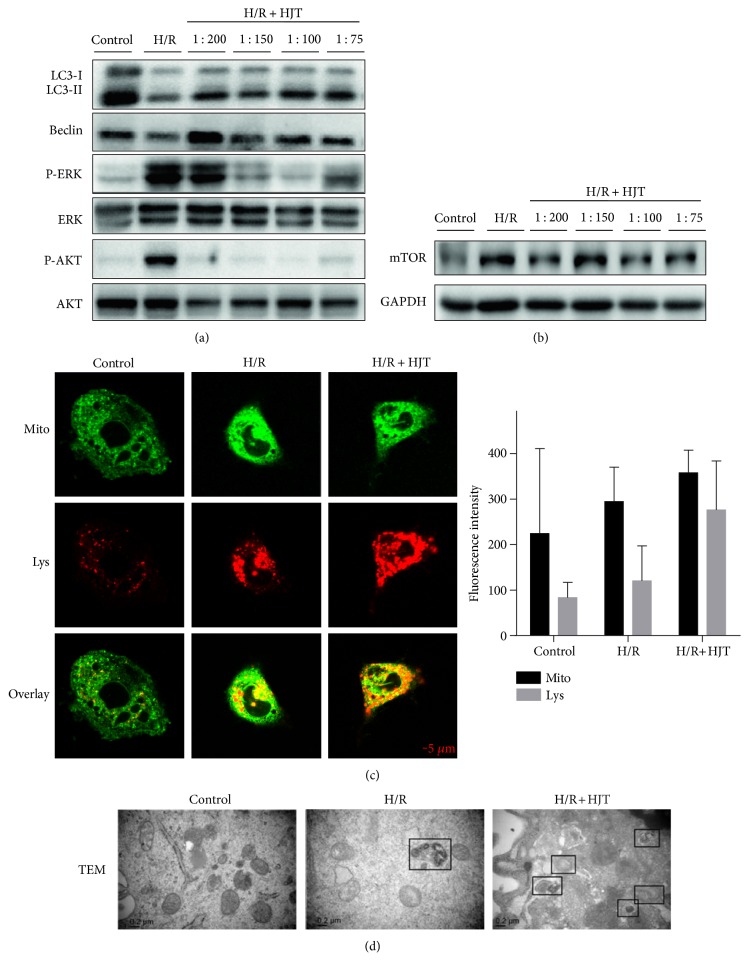
HJT promoted autophagic flux in H/R-induced H9c2 cardiomyocytes. (a) Western blot analysis of autophagy-related proteins: LC3-I, LC3-II, Beclin, P-ERK, ERK, P-AKT, and AKT. H9c2 cardiomyocytes were subjected to hypoxia for 24 h and reoxygenation for 2 h. (b) Protein expression levels of mTOR was determined by Western blot analysis. (c) After treatment with hypoxia for 12 h followed by 2 h reoxygenation, H9c2 cardiomyocytes were loaded with MitoTracker Green (1 *μ*M) and LysoTracker Red (1 *μ*M). Images were captured by confocal microscopy, scale bar, 5 *μ*m. (d) Electron micrographs of cardiomyocytes in different groups (50000×). Black boxes indicate autophagosomes; scale bar, 0.2 *μ*m.

It was reported that several pathways regulate both autophagic and apoptotic machinery, while autophagy can cooperate with apoptosis [31]. Autophagy as a cell survival mechanism delays apoptotic cell death following DNA damage [32]. Therefore, we determined whether or not HJT could induce autophagy in H9c2 cardiomyocytes exposed to H/R. We measured the protein levels of LC3-I, LC3-II, Beclin, mTOR, AKT, and ERK to evaluate whether HJT was able to activate the autophagy-related signaling pathway in H9c2 cardiomyocytes. As shown in Figure 4(a), the expressions of LC3-II and Beclin, two marker proteins of autophagy, were decreased in the H/R-induced group. Conversely, the HJT treatment can increase the expressions of LC3-II and Beclin in cardiomyocytes with H/R injury. PI3K/AKT and MEK/ERK are two major pathways that can activate autophagy. According to the results of Western blotting, it is clear that the H/R-induced increases of phos-ERK and phos-AKT expression were inhibited by the HJT treatment. Moreover, the protein level of mTOR was increased in the H/R group and decreased in the HJT groups (Figure 4(b)). These data above suggested that HJT could facilitate autophagy in H/R-induced H9c2 cardiomyocytes.

For more direct evidences to verify the assumption above, mitochondria and lysosomes were visualized with fluorescent MitoTracker Green and LysoTracker Red [33], and TEM were also performed [34]. In the control cells, few mitochondria and lysosomes with fluorescent labels were visualized while fluorescent increased in the H/R and HJT cell groups (Figure 4(c)). In addition, TEM assay confirmed the accumulation of autophagosomes with double-membraned structures in H/R-induced H9c2 cardiomyocytes, and the changes were further promoted in the HJT treatment group (Figure 4(d)).

### 3.5. HJT Decreased Myocardial Injury and Improved the Cardiac Function in MI Rats

**Figure 5:**
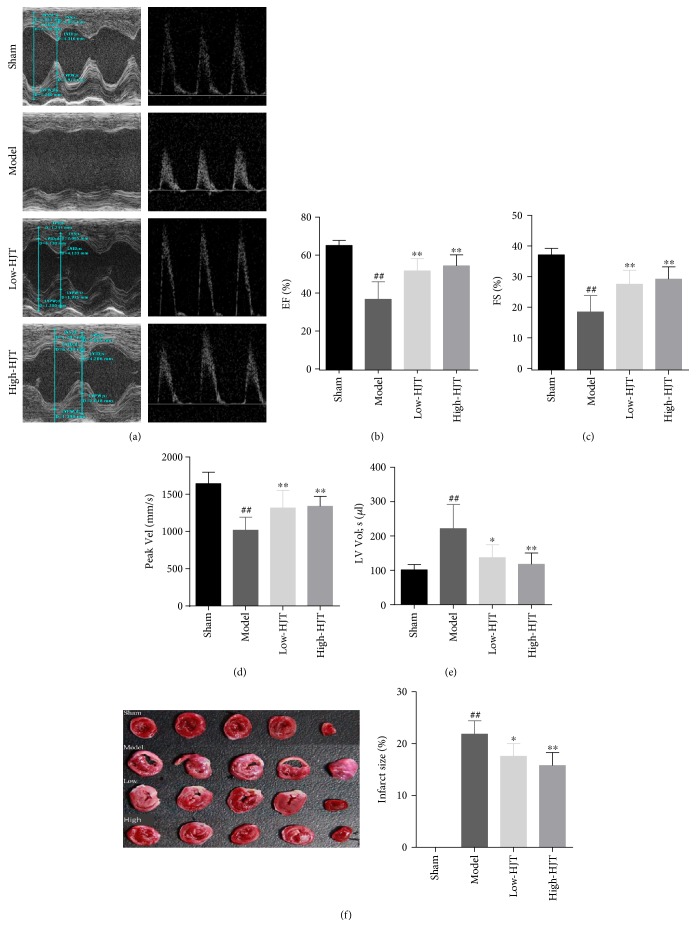
Protective effect of HJT on experimental myocardial ischemia in rats. (a) Representative images of echocardiography among the different groups. (b–e) Evaluations of the left ventricular ejection fraction (EF %) (b), fractional shortening (FS %) (c), average peak velocity (Peak Vel) (d), and left ventricular systolic volume (e). (f) Representative images of rat heart slices and quantification of heart infarct size in the different groups (*n* = 5). ^##^*P* < 0.01 versus the sham group, ^∗^*P* < 0.05, and ^∗∗^*P* < 0.01 versus the group treated with LAD ligation alone.

**Figure 6:**
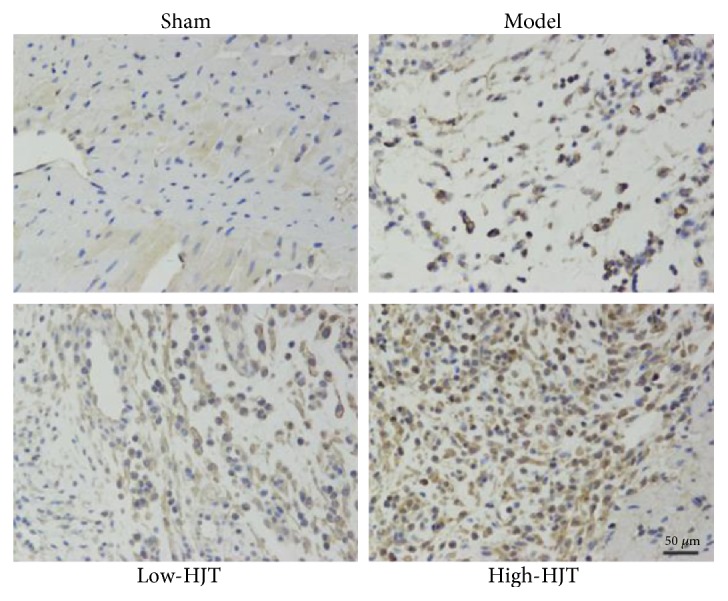
The expression of LC3B was assayed by immunohistochemical staining to analyze cell autophagy, scale bar, 50 *μ*m.

To verify that HJT could reverse the damage of cardiac function caused by MI, rats underwent coronary artery ligation for 7 days, and subsequently echocardiography and hemodynamic measurements were further performed. As shown in Figures 5(a), 5(b), 5(c), 5(d), and 5(e), HJT upgraded the value of EF%, FS % and Peak Vel in MI hearts and decreased the value of LV volumes significantly. No infarct area was observed in the sham group while noticeable infarct areas were observed in the model group as evidenced by a large TTC negatively stained area (Figure 5(f)). Both the low- and high-dose HJT treatment groups significantly decreased the infarct area compared with the model group (21.84 ± 2.58, 17.56 ± 2.45, and 15.78 ± 2.51 in model, low dose, and high dose of HJT, resp.). The LC3B is an important marker of autophagy, which is in accordance to previous report [35]. MI hearts showed a significant accumulation of LC3B positive cells, which was further promoted by HJT (Figure 6()). Collectively, these data suggested that the HJT treatment reduced infarct size and improved myocytes viability and cardiac performance in a rat model of MI injury by promoting autophagy.

## 4. Discussion

In the present study, we describe an antioxidation injury ability of HJT by regulation the balance of autophagy and apoptosis. Our results support a pathway wherein AKT and ERK might be involved in the HJT-induced Beclin-1 and mTOR-dependent autophagy. Thus, our results underscore a regulation balance of autophagy and apoptosis by HJT in myocardial oxidative damage. Autophagy and apoptosis are two self-destructive processes and responsible for a variety of cellular homeostasis functions. The cytoprotective function of autophagy is widely accepted in mediating negative modulation of apoptosis in many circumstances especially in cardiovascular diseases [31]. Cardiomyocyte autophagy is essential for maintaining cellular function and survival; lack of autophagy may lead to cardiac hypertrophy, left ventricular dilatation, and contractile dysfunction. Therefore, autophagy is activated to exert a protective role on the cardiomyocytes by reducing levels of oxidative damage [36]. In this study, we found that HJT protected H9c2 cardiomyocytes from H/R-induced oxidative stress injury by improving H9c2 cardiomyocytes autophagy and inhibiting apoptosis. Cardiomyocyte autophagy was also observed in the MI model after the HJT treatment with the improvement of cardiac function. Autophagy induced by HJT might lead to upregulated mitochondrial oxygen consumption, increased ATP level, and decreased intracellular ROS level and MDA content.

Autophagy is rapidly regulated by nutrient starvation, growth factor withdrawal, or oxidative damage; the mechanisms mediating the complex regulation of apoptosis and autophagy are not yet fully understood. The mammalian autophagy gene Beclin-1 is important for localization of autophagic proteins to a pre-autophagosomal structure, depending on interaction with the class III-type phosphoinositide 3-kinase (PI3KC3). Activation of the PI3K-Akt-mTOR signaling pathway can promote necrotic cell death via suppression of autophagy [37]. In our study, we found that H/R resulted in an increase in P-AKT/AKT ratio and a decrease in Beclin-1, and HJT treatment decreased the ratio of P-AKT/AKT and improved the level of Beclin-1. All HJT-treated groups showed rising trends of LC3-II compared to the H/R group. In addition, Bcl-2 binds to Beclin-1 and inhibits autophagy [38]; it was not observed in our results. These data suggest that AKT/Beclin-1 pathway may play an important role in HJT-induced autophagy after myocardial oxidative damage.

mTOR is a major negative regulator of autophagy. It can be activated by PI3K/AKT and MEK/ERK [39, 40], then negatively regulates the activity of the autophagy-initiation kinase ULK1 complex via phosphorylation [41]. Our results further confirmed that phosphorylation of ERK and mTOR protein level were increased after H/R, and the HJT treatment downregulated the ratio of P-ERK/ERK and mTOR expression. There was a dose-dependent trend of decrease of P-ERK/ERK ratio compared to that of the H/R group from the WB band. And considering our evidences of histological and TEM results, we can get a conclusion that HJT can promote autophagy. These findings may suggest that mTOR pathways coregulated by AKT and ERK were also involved in HJT-induced autophagy after myocardial oxidative damage.

## 5. Conclusion

In summary, our study demonstrates that HJT upregulated the viability, ATP level, and mitochondrial oxygen consumption of cardiomyocyte and improved the cardiac function in MI rats by promoting the autophagy and inhibiting the apoptosis. We provide evidence that the mechanism involves activation of AKT/Beclin-1, AKT, and ERK/mTOR pathway in cardiomyocyte autophagy. The work provides new mechanistic insight into HJT therapy and places cardiomyocyte autophagy as a key factor that improves the cardiac function after MI.

## Abbreviations

HJT: Hongjingtian Injection
H/R: Hypoxia/reoxygenation
MI: Myocardial ischemia.
